# Suppressed activity of the rostral anterior cingulate cortex as a biomarker for depression remission

**DOI:** 10.1017/S0033291721004323

**Published:** 2023-04

**Authors:** Christopher G. Davey, Micah Cearns, Alec Jamieson, Ben J. Harrison

**Affiliations:** 1 Department of Psychiatry, The University of Melbourne, Melbourne, Australia; 2 Discipline of Psychiatry, School of Medicine, The University of Adelaide, Adelaide, Australia

**Keywords:** Depression, treatment, remission, fMRI, machine learning

## Abstract

**Background:**

Suppression of the rostral anterior cingulate cortex (rACC) has shown promise as a prognostic biomarker for depression. We aimed to use machine learning to characterise its ability to predict depression remission.

**Methods:**

Data were obtained from 81 15- to 25-year-olds with a major depressive disorder who had participated in the YoDA-C trial, in which they had been randomised to receive cognitive behavioural therapy plus either fluoxetine or placebo. Prior to commencing treatment patients performed a functional magnetic resonance imaging (fMRI) task to assess rACC suppression. Support vector machines were trained on the fMRI data using nested cross-validation, and were similarly trained on clinical data. We further tested our fMRI model on data from the YoDA-A trial, in which participants had completed the same fMRI paradigm.

**Results:**

Thirty-six of 81 (44%) participants in the YoDA-C trial achieved remission. Our fMRI model was able to predict remission status (AUC = 0.777 [95% confidence interval (CI) 0.638–0.916], balanced accuracy = 67%, negative predictive value = 74%, *p* < 0.0001). Clinical models failed to predict remission status at better than chance levels. Testing the model on the alternative YoDA-A dataset confirmed its ability to predict remission (AUC = 0.776, balanced accuracy = 64%, negative predictive value = 70%, *p* < 0.0001).

**Conclusions:**

We confirm that rACC activity acts as a prognostic biomarker for depression. The machine learning model can identify patients who are likely to have difficult-to-treat depression, which might direct the earlier provision of enhanced support and more intensive therapies.

## Introduction

It is anticipated that brain imaging technologies will one day help the mental health clinician choose the treatment that is most likely to benefit the patient before them. However, such treatment biomarkers, imaging and otherwise, have yet to have been adopted in psychiatric care. Treatments for depressed patients are usually offered sequentially, and amended using a trial-and-error approach. A reliable biomarker for treatment response could act to accelerate the delivery of effective treatments.

Early nuclear imaging studies reported that increased resting-state activity in the rostral anterior cingulate cortex (rACC) predicted response to antidepressant medications (Brannan et al., 2000; Mayberg et al., 1997; Saxena et al., 2003). Similar findings have been reported in studies that have used functional magnetic resonance imaging (fMRI) (Keedwell et al., 2010; Langenecker et al., 2007; Meyer et al., 2019Sikora et al., 2016; ) and electroencephalography (EEG) (Korb, Hunter, Cook, & Leuchter, 2011; Pizzagalli et al., 2001, 2018). Increased rACC activity has also predicted response to sleep deprivation (Wu et al., 1999; Clark et al., 2006), transcranial magnetic stimulation (TMS) (Drysdale et al., 2017; Kito, Fujita, & Koga, 2008), and electroconvulsive therapy (ECT) (McCormick et al., 2007). Rather than predicting response to a particular treatment, these results suggest that rACC activity predicts the overall likelihood of improvement irrespective of the type of treatment provided.

### The rostral anterior cingulate cortex and depression

The rACC is a component of the default mode network (DMN), a set of regions that is typically active during self-directed thought (including at rest) and suppressed during engagement with attentionally demanding tasks (Harrison et al., 2008). Depressed participants show weaker rACC suppression than healthy control participants during the performance of such tasks (Rose, Simonotto, & Ebmeier, 2006; Wagner et al., 2006); with their failure to suppress rACC activity predicting poor treatment response (Keedwell et al., 2010). It has been suggested that altered rACC activity in depression reflects maladaptive self-related patterns of thought, and that difficulties in suppressing such processes indicate a more entrenched depression that is more difficult to treat (Pizzagalli, 2011).

Most of the studies that have examined rACC activity as a treatment biomarker have been open-label studies, where all participants have received the same treatment. Such designs do not allow for the determination of the predictive *v.* prognostic biomarker status of rACC activity (i.e. whether it predicts response to a particular treatment or the overall likelihood of improvement). This can only occur in studies that include multiple treatments, and preferably where participants have been randomised to them. In one of the few studies to have done so (the EMBARC trial), patients underwent baseline fMRI and EEG before being randomised to treatment with either sertraline or placebo (Trivedi et al., 2016). The analysis of EEG data showed that increased rACC theta activity was prognostic, predicting improvement in depressive symptoms in both treatment groups (Pizzagalli et al., 2018). Secondary analysis of fMRI data showed that rACC activity suppression during an emotional conflict task also predicted outcome across both groups (Fonzo et al., 2019).

### Using machine learning to characterise biomarker performance

Machine learning approaches in neuroimaging have permitted a more detailed examination of the utility of putative biomarkers (Cearns, Hahn, & Baune, 2019; Janssen, Mourão-Miranda, & Schnack, 2018). Whereas conventional approaches typically characterise group-averaged effects, machine learning models can be trained to provide optimal classification accuracy for the individual patient: for example, determining the likelihood of the patient responding to particular treatments (as for a predictive biomarker) or of having a favourable prognosis irrespective of treatment (for a prognostic biomarker). For neuroimaging to demonstrate practical clinical utility, it is essential that the characteristics of an imaging biomarker are fully described, including details on its sensitivity, specificity, and predictive value (Gillan & Whelan, 2017).

In this study, we sought to determine whether weaker rACC activity suppression during task performance was a prognostic biomarker, and one that had utility at the individual patient level. We trained and tested support vector machine (SVM) models on data from our recently published clinical trial of cognitive behavioural therapy (CBT) plus fluoxetine or placebo for youth depression (the YoDA-C trial) (Davey et al., 2019). Prior to commencing treatment, participants had completed an fMRI task that reliably produces suppression of rACC activity (Harrison et al., 2011). We further tested our model on an fMRI dataset from an accompanying clinical trial that examined the use of anti-inflammatory medications for youth depression (the YoDA-A trial) (Berk et al., 2020). Participants had completed the same fMRI task before being randomised to the addition of aspirin, rosuvastatin, or placebo to treatment-as-usual.

Our hypothesis was that weaker suppression of the rACC during task performance would predict failure to remit to treatment at the individual level, irrespective of which of the five treatments the participant had received across the two trials. We anticipated that this ability to predict outcome using brain imaging data would be superior to prediction using only clinical and behavioural variables. Our study included young patients who had illnesses that were relatively severe, but at early stages and therefore less complicated by chronicity and treatment failure. Our aim was to characterise the performance of rACC activity suppression as a prognostic biomarker, and to determine whether its accuracy was sufficient for clinical utility.

## Methods

### Participants

Participants for our training and validation analyses were 15- to 25-year-olds with moderate-to-severe MDD. They were enrolled in the YoDA-C clinical trial, which compared treatment with CBT and fluoxetine with treatment with CBT and placebo. The study was approved by the Melbourne Health Human Research Ethics Committee (HREC/12/MH/151 and HREC/12/MH/255) and was monitored by a Data Safety and Monitoring Board. All procedures contributing to this work complied with the ethical standards of the relevant national and institutional committees on human experimentation and with the Helsinki Declaration of 1975, as revised in 2008. All participants provided their written informed consent, which was also obtained from a parent or legal guardian if the participant was younger than 18 years of age. Inclusion and exclusion criteria are listed in the supplementary materials.

Of the 153 young people who participated in the YoDA-C trial, 105 consented to undergo optional baseline neuroimaging before commencing the trial treatments. Of these, seven were excluded from our analyses because they did not return for post-baseline assessment, eight due to excessive head movement during fMRI, one due to an incidental brain pathology finding, and eight due to poor task performance (see below for further details). Data from 81 participants were included in the analyses. We further tested our model on a sample of 25 participants, each of whom had participated in the YoDA-A trial and completed the same imaging protocol. This data was used only for validation, and was not used at all in the development of the classifier.

### Trial design

The YoDA-C study was a 12-week, parallel-group, double-blind randomised control trial in which participants with moderate-to-severe MDD were allocated to treatment with either CBT and fluoxetine or CBT and placebo (Davey et al., 2019). Participants were commenced on one 20-mg capsule of fluoxetine or one placebo pill. The medication could be increased to fluoxetine 40 mg daily (or two placebo pills) if there was an insufficient clinical response at any time after the first 4 weeks. All participants were offered manualised CBT, delivered by therapists in weekly 50-min sessions.

Participants attended interviews at baseline, and at weeks four, eight and 12, during which they completed assessments with research assistants. They saw a psychiatrist or psychiatry trainee to complete medical assessments at the same time-points. The primary outcome measure was the Montgomery-Åsberg Depression Rating Scale (MADRS) (Montgomery & Asberg, 1979), an interviewer-rated measure of depression severity, with a change in a score at 12 weeks as the primary outcome.

As reported in the trial manuscript, there was no difference between the groups for change in MADRS score at 12 weeks, and nor for rates of remission (Davey et al., 2019). For our machine learning analyses, we examined remission as our outcome of interest. We considered a patient to be in remission with a MADRS score ⩽12 at their last post-baseline assessment; a commonly used definition (e.g. Nierenberg, Feighner, Rudolph, Cole, & Sullivan, 1994; Popova et al., 2019), and one that provided approximately balanced groups for remission *v.* non-remission (44% *v.* 56%). We also performed analyses with other definitions of remission: for MADRS ⩽7 (the definition used in our trial paper) and for MADRS ⩽10. We also examined treatment response as an outcome, defined as a change in MADRS scores of 50% or greater by week 12.

In the accompanying YoDA-A trial, participants were treated with rosuvastatin 10 mg daily, aspirin 100 mg daily, or placebo in addition to treatment-as-usual (Berk et al., 2020). They completed the same assessments at the same timepoints as those in the YoDA-C trial. There were also no significant differences between groups for change in MADRS scores or for other depression-related outcomes in this trial.

### Imaging task and the preparation of first-level maps

Participants completed an fMRI emotional face-matching task before commencing the trial treatments. In brief, the task required participants to match the gender of a face displaying an emotional expression at the top half of the screen to the gender of one of two faces showing the same expression in the bottom half of the screen. A control task required the matching of shapes. Details of the task, image acquisition, and image preprocessing steps are listed in the supplementary materials.

The task was modelled by specifying primary regressors for the shape and face matching conditions, followed by convolution with a canonical hemodynamic response function. Parameter estimates were calculated at each voxel using the general linear model, adjusted for motion. Our contrast of interest was shapes > faces, which captures the suppression of activity that occurs routinely in rACC during engagement with face-matching tasks (Harrison et al., 2011). A single first-level contrast image was created for each participant for inclusion in machine learning analyses, and was also entered into group-level analyses to illustrate whole-brain task effects (voxel-wise corrected using *P*_FDR_ < 0.01).

To restrict our focus to rACC activity, we defined an independent region of interest (ROI) based on a meta-analytic search (using the term ‘default mode’) of 907 studies in the Neurosynth database (Yarkoni, Poldrack, Nichols, Van Essen, & Wager, 2011). We defined our ROI as a 5 mm radial sphere centred on the peak voxel in the anterior DMN component from the Neurosynth map (MNI coordinates: *x* = −2, *y* = 50, *z* = −6). Note that this region is described in other contexts as being in the ventromedial cortex: we describe it as rACC to maintain consistency with previous studies. The 5 mm ROI contained 81 voxels. We also assessed the predictive capacity of a larger 10 mm ROI (515 voxels). The fMRI data were prepared for our machine learning pipeline using Nilearn (Abraham et al., 2014), which parsed the participants’ contrast images (NIfTI files) into one-dimensional arrays.

### Clinical models

In addition to training an fMRI model on rACC activity suppression, we also trained comparison models constructed from clinical data to determine what, if any, improvements our imaging model would confer. Our primary clinical model was trained using age, sex, the severity of baseline depressive symptoms (measured with the MADRS (Montgomery & Asberg, 1979) and Quick Inventory of Depressive Symptomatology [QIDS] (Rush et al., 2003)), the severity of baseline anxiety symptoms (assessed with the Generalised Anxiety Disorder 7-item questionnaire [GAD-7] (Spitzer, Kroenke, Williams, & Löwe, 2006)), number of major depressive episodes, duration of the present episode, and trial treatment allocation (CBT and fluoxetine or CBT and placebo). To ensure that other discriminative clinical data were not overlooked, we trained a second exploratory clinical model using the elastic net method for feature selection (Zou & Hastie, 2005), with selected features passed into a linear SVM. This model included all of the demographic, clinical, and psychometric measures that were collected for the trial (see the online Supplementary Table S1 for a list of included features).

### Machine learning pipeline

The SVM models were fitted used sklearn (Pedregosa et al., 2011) in Python 3.6, which included training and testing using nested cross-validation (Varoquaux et al., 2017). We used an outer 10-fold cross-validation loop to approximate model performance separate from all data transformations and feature selection (Cawley & Talbot, 2010; Varoquaux et al., 2017). For the fMRI model, we used independent components analysis (ICA) to reduce the dimensionality of the voxel space before passing them into a linear SVM. For the primary clinical model, given that the number of clinical predictors was already low, we included all eight predictors, while for the secondary clinical model we used the elastic net to isolate the most predictive subset. Inner loop cross-validation, using five repeats of 10 folds, was performed via the following steps. First, we standardised all predictors to have a mean of zero and a standard deviation of one. Next, due to class imbalance in the outcome labels (56% *v.* 44%) we over-sampled the minority class (remission) using synthetic minority over-sampling (SMOTE) (Chawla, Bowyer, Hall, & Kegelmeyer, 2002). To avoid selecting ICA components and SVM hyperparameters based on site-specific distributions in the inner cross-validation loop, we used a grid-search with leave site out cross-validation (LSO). Specifically, model hyperparameter combinations were trained on one data collection site and tested on the others in an iterative fashion to find the most generalisable subset for final testing in the outer cross-validation loop (Cearns et al., 2019).

**Fig. 1.**
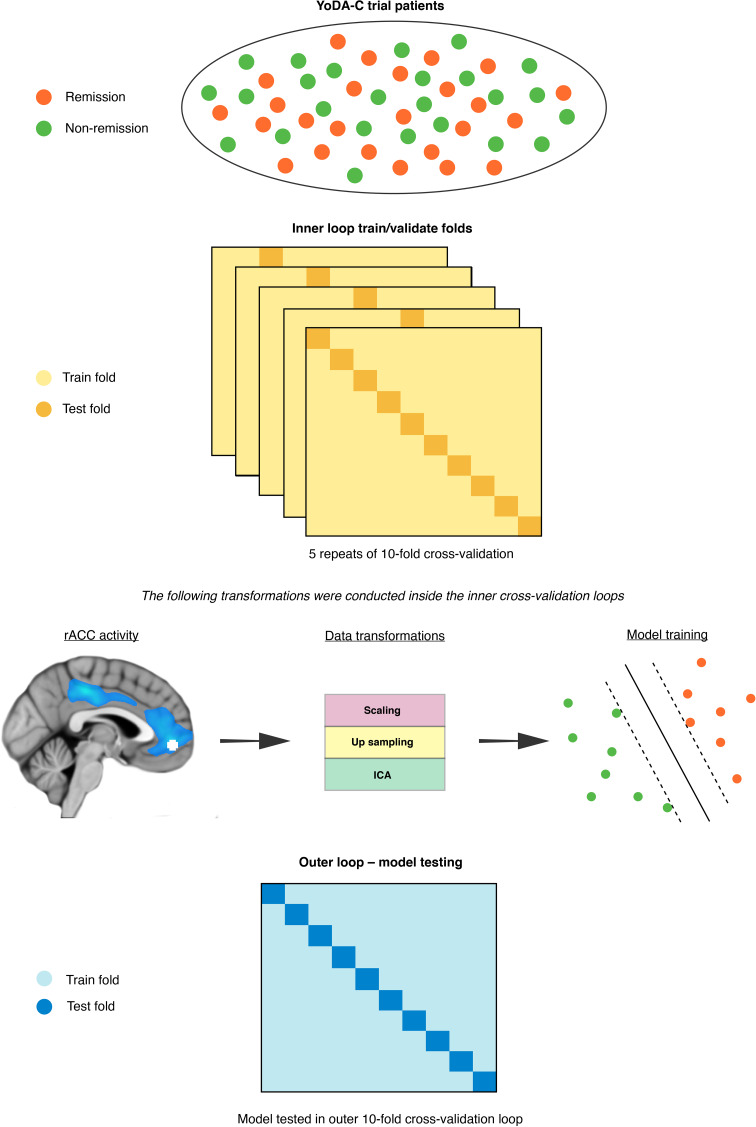
The machine learning pipeline. The nested cross-validation scheme incorporated an inner loop, within which the imaging data were transformed before SVMs were trained, and a final outer 10-fold cross-validation loop where each SVM was tested.

We selected the set of independent components and values for the L1 ratio, alpha, and C that maximised area under the curve (AUC) on the receiver operator characteristic. We then used Platt scaling to calibrate the predicted probabilities of the SVM inside the cross-validation folds (Cearns, Hahn, Clark, & Baune, 2020). This method takes the final weighted values for the trained SVM and uses logistic regression to map probability estimates to each remission label. Finally, we used permutation tests to assess the statistical significance of our final models (*m* *=* 10 000) (Ojala & Garriga, 2010). We determined a significant omnibus effect between models using a Kruskal-Wallis H-test. Following, we used Mann-Whitney rank tests to assess for post hoc differences between our three models and corrected *p*-values using the false discovery rate. The machine learning pipeline is illustrated in Fig. 1. Analysis code is available on request. All final trained models are available at the Photon AI online model repository (https://www.photon-ai.com/repo).

## Results

### Participant characteristics and treatment outcome

The 81 participants whose fMRI data were used in the machine learning analyses were a subset of the 153 participants who participated in the YoDA-C trial: the participants who were included in the analyses did not differ on any baseline characteristic from participants who did not (online Supplementary Table S2). The participants were randomised to receive either CBT and fluoxetine (*n* = 44) or CBT and placebo (*n* = 37). They had a mean age of 19.8 (s.d., 2.7) years, and had severe depression, with a mean MADRS score of 32.6 (s.d., 5.5). Both treatment groups showed a reduction in MADRS scores over the course of the trial [−15.7 [95% confidence interval (CI) −19.0 to −12.4] in the CBT and placebo group and −14.5 (95% CI −17.7 to −11.4) in the CBT and fluoxetine group]. As in the larger trial, there was no significant between-group difference for change in MADRS scores [1.2 (95% CI −3.2 to 5.5), *p* = 0.59]. Nor was there a difference between rates of remission (defined as MADRS ⩽ 12), with 15 of 37 (41%) achieving remission in the CBT and placebo group and 21 of 44 (48%) in the CBT and fluoxetine group [OR 1.3 (95% CI 0.5–3.6), *p* = 0.65]. The baseline characteristics of the remission and non-remission groups are listed in Table 1, with further details of treatment outcomes listed in online Supplementary Table S3. The baseline characteristics of participants in the YoDA-A trial, which comprised the second test dataset, are detailed in online Supplementary Table S4 and their response to treatment in online Supplementary Table S5.

**Table 1.** Baseline characteristics of the participants

	Descriptive statistic	Remission	Non-remission	Remission *v*. non-remission
Baseline characteristic		*N* = 36	*N* = 45	*p* value^a^
Age	Mean (s.d.)	19.3 (2.7)	20.1 (2.6)	0.23
Sex, female	*N* (%)	22 (61%)	29 (64%)	0.82
MADRS score	Mean (s.d.)	31.3 (5.0)	33.6 (5.7)	0.06
QIDS score	Mean (s.d.)	16.0 (3.7)	17.7 (2.4)	0.02
GAD7 score	Mean (s.d.)	12.0 (5.3)	14.2 (5.2)	0.06
Number of major depressive episodes	Median (IQR)	2 (1–4)	3 (1–5+)	0.17
Duration, weeks	Median (IQR)	16 (11–46)	25 (11–54)	0.50
Treatment allocation, CBT and fluoxetine	*N* (%)	21 (58%)	23 (51%)	0.65

a Calculated using *t* tests for normally distributed variables, Wilcoxon tests for skewed variables, and Fisher's exact test for categorical variables.

### Behavioural and imaging data

Behavioural data from the fMRI task confirmed that response times (RTs) for the faces were longer than for the shapes (faces RT = 1.32 s, shapes RT = 0.81 s, *p* < 0.001), although levels of accuracy were similar (faces = 96%, shapes = 97%). As expected, the primary fMRI contrast of interest (shapes > faces) showed significant suppression of activity in extended DMN regions, including the anterior midline cortex, encompassing the rACC (online Supplementary Fig. S1 and Table S6).

### Prediction of treatment outcome

The SVM for the fMRI model of rACC activity suppression achieved a moderate and statistically significant level of outer cross-validated accuracy [test AUC = 0.777, (95% CI 0.638–0.916), *p* = <0.0001], using four independent components selected in the inner cross-validation loop (Table 2, online Supplementary Fig. 2). The primary clinical model, which comprised eight hypothesis-driven predictors, did not perform better than chance [test AUC = 0.505 (95% CI 0.402–0.618), *p* = 0.48]. Our exploratory clinical model, which used the elastic net variable selection technique, selected five variables for prediction: the MADRS items for inability to feel, reduced sleep, and pessimistic thoughts; and the Cognitive Emotion Regulation Questionnaire (CERQ) (Garnefski, Kraaij, & Spinhoven, 2001) items for self-blame and putting things into perspective. This model also performed at no better than chance [test AUC = 0.613 (95% CI 0.466–0.727), *p* = 0.23]. Significant differences were found between models after correcting for ties (*H* = 8.9, *p* = 0.01), with the fMRI model significantly outperforming both the primary (*p* = 0.006) and exploratory clinical models (*p* = 0.018), demonstrating significant incremental utility. The results for fMRI models that used a larger (10 mm) rACC ROI and alternative definitions of remission (MADRS ⩽ 10 and MARDS ⩽ 7) and response are presented in Table 3.

**Table 2.** Performance metrics for all classifiers

	Train AUC (95% CI)	Test AUC (95% CI)	F1	BAC	Acc	Sens	Spec	PPV	NPV	*p* value
*Nested SVM results from the YoDA-C study*										
fMRI SVM	0.789 (0.775–0.803)	0.777 (0.638–0.916)	64.9%	67.0%	65.9%	77.5%	56.5%	61.8%	74.0%	<0.0001
Clinical SVM (*primary*)	0.684 (0.380–0.629)	0.505 (0.402–0.618)	51.1%	52.0%	53.2%	40.0%	64.0%	53.3%	58.9%	0.48
Clinical SVM (*exploratory*)	0.848 (0.473–0.753)	0.613 (0.446–0.727)	49.2%	52.1%	51.3%	56.7%	47.5%	50.3%	61.3%	0.23
*Independent validation of fMRI SVM results in the YoDA-A study*										
fMRI SVM	–	0.776	63.7%	64.4%	64.0%	75.0%	53.9%	60.0%	70.0%	<0.0001

AUC, Area-under-the-Curve; F1, Harmonic mean of sensitivity and specificity; BAC, Balanced accuracy; Acc, Accuracy; Sens, sensitivity; Spec, Specificity; PPV, Positive predicted value; NPV, Negative predicted value.

**Table 3.** Performance metrics for the fMRI classifier using different definitions of remission, response, and rACC ROI sizes

	Train AUC (95% CI)	Test AUC (95% CI)	F1	BAC	Acc	Sens	Spec	PPV	NPV
*5 mm mask*									
MADRS ⩽ 7	0.722 (0.705–0.740)	0.701 (0.602–0.801)	55.3%	57.9%	54.2%	65.0%	50.7%	29.2%	79.3%
MADRS ⩽ 10	0.770 (0.746–0.794)	0.727 (0.523–0.925)	61.0%	63.3%	61.5%	70.0%	56.7%	55.2%	70.5%
MADRS ⩽ 12	0.789 (0.775–0.803)	0.777 (0.638–0.916)	64.9%	67.0%	65.9%	77.5%	56.5%	61.8%	74.0%
MADRS ⩽ 50%	0.815 (0.796–0.834)	0.832 (0.728–0.936)	70.6%	72.3%	72.0%	76.7%	68.0%	73.4%	79.3%
*10 mm mask*									
MADRS ⩽ 7	0.737 (0.721–0.753)	0.726 (0.582–0.871)	50.6%	62.0%	50.4%	85.0%	39.0%	32.9%	83.3%
MADRS ⩽ 10	0.786 (0.770–0.806)	0.680 (0.478–0.882)	51.5%	59.3%	54.0%	80.0%	38.7%	44.4%	69.2%
MADRS ⩽ 12	0.811 (0.797–0.824)	0.792 (0.658–0.925)	67.2%	69.8%	68.4%	82.5%	57.0%	61.0%	82.5%
MADRS ⩽ 50%	0.831 (0.819–0.844)	0.843 (0.751–0.936)	73.1%	74.8%	74.4%	89.2%	60.5%	69.3%	85.8%

AUC, Area-under-the-Curve; F1, Harmonic mean of sensitivity and specificity; BAC, Balanced accuracy; Acc, Accuracy; Sens, sensitivity; Spec, Specificity; PPV, Positive predicted value; NPV, Negative predicted value.

The fMRI model showed a sensitivity of 77.5%, and a specificity of 56.5%. The negative predictive value (NPV) of the model (the probability of correctly predicting that a young person would not remit) was 74.0%, indicating the potential clinical utility of the classification for predicting the likelihood of non-remission. The fMRI model performed similarly in the independent test set from the YoDA-A trial (test AUC 0.776, *p* < 0.0001). The model's sensitivity was 75.0%, specificity 53.9%, and NPV 70.0% (Table 2).

## Discussion

Our study confirms the utility of rACC activity as a prognostic imaging biomarker for depression. We showed that weaker suppression of rACC during task engagement predicted the likelihood of non-remission after 12 weeks of treatment. The fMRI classifier performed better than clinical classifiers, suggesting its potential utility for clinicians. The classifier's prediction of non-remission was correct for 70% to 74% of patients (across both trial samples). This was true for participants who were to commence a diverse range of treatments, indicating that the predictive utility of rACC function is not treatment specific (i.e. it acts as a prognostic rather than predictive biomarker). This result adds to similar conclusions from the recent analysis of EEG and fMRI data from the EMBARC trial (Fonzo et al., 2019; Pizzagalli et al., 2018), extending their findings to depressed youth, and detailing the predictive characteristics of rACC suppression.

A prognostic biomarker has potential value to clinicians, who do not accurately predict the likelihood of response from clinical impressions alone (Chekroud et al., 2016; Leuchter et al., 2009). The prediction that a patient is unlikely to remit in the short term can act to direct a clinician's attention to the fact that the depression will be difficult to treat at an early stage, prior to the patient enduring multiple treatment failures. It suggests the patient might require close monitoring and additional supports, along with the accelerated ‘stepping up’ of treatments. Conversely, where the classifier suggests a likelihood of a good outcome, more benign treatments such as watchful waiting or supportive psychotherapy might be suggested initially.

We used an attentionally demanding task that reliably suppressed rACC activity. The suppression of rACC activity is hypothesised to reflect the suppression of affective and self-related mental content that might interfere with task engagement (Harrison et al., 2011). Our task was not only attentionally demanding, but it incorporated the implicit processing of affectively laden facial expressions. One interpretation is that negative emotional expressions intrude into the attentional space of susceptible participants via their self-related characteristics, preventing suppression of rACC activity. This is consistent with the decreased theta wave activity that has been shown to predict the poor outcome – it is associated with decreased ability to focus attention (Asada, Fukuda, Tsunoda, Yamaguchi, & Tonoike, 1999). It is not yet clear why weaker suppression of the rACC characterises difficult-to-treat depression: whether this difficulty is related to the intrusive nature of self-related affective stimuli or to more basic processes. Notwithstanding some uncertainty about the underlying mechanisms, there is nonetheless now good evidence that the extent of rACC suppression during task performance is important for understanding the longer-term trajectory of depression.

We used the same machine learning approach to examine whether an SVM classifier could be trained on clinical parameters. In our primary model, which used clinical parameters that have previously been shown to predict depression outcome, the classifier failed to perform at a better than chance level. Clinical factors have shown only weak predictive power in large samples (Chekroud et al., 2016; Uher et al., 2012), and their failure to show utility at the individual level in our study might be due to our modest sample size. To address concerns that have been raised in prior imaging machine learning studies – that the clinical model had been designed to fail and to inflate the relative success of the imaging model (Nelson, Yung, & McGorry, 2019) – we additionally tested a model that included all available demographic, clinical and psychometric data. It too failed to classify patients beyond chance.

It is important to note that in our analyses, where baseline clinical data provided no information to inform classification, a model that includes both the imaging data and baseline clinical data will perform the same as a model that includes only the imaging data. This reinforces the difficulties that clinicians have had in finding useful clinical markers to inform treatment outcome. While in this study the clinical data did not add useful information, if we can better understand the brain basis for the role that rACC suppression has in predicting outcome, we might better integrate imaging and clinical data to improve predictive modelling.

Our total clinical sample of 106 participants is one of the largest fMRI studies of rACC biomarker status. While we used a larger sample than most, it is still a relatively small sample from a machine learning perspective, with a consequent risk of systematic overestimation of the classifiers’ accuracy. It has been argued that higher accuracy estimates for small samples arise due to sample homogeneity, while larger samples tend to yield more modest estimates due to increased sample heterogeneity (Schnack & Kahn, 2016). Overall, however, by using two levels of validation (outer 10-fold cross-validation and across-study validation), we believe that the probability of sample size-based accuracy overestimation is low. The further testing of our model in the separate trial did occur in participants who were recruited from overlapping clinical settings, and who completed the same fMRI task on the same scanner: more diverse samples will be needed to define the bounds of model performance (Hahn, Ebner-Priemer, & Meyer-Lindenberg, 2019). While the patients in the trials were relatively young, and at early stages of illness, the relevance of rACC suppression for predicting treatment outcomes is consistent with findings from older patient groups.

Altered rACC activity has shown promise as a potential biomarker for treatment outcome in depression after first being identified two decades ago, but its characteristics as a predictor at the individual patient level have until now remained uncertain. Our study suggests that weaker suppression of rACC activity during an attentionally demanding affective task might be particularly useful for identifying patients who are likely to have difficult-to-treat illnesses. The biomarker was less useful at identifying patients with good prognoses (its specificity and positive predictive value were relatively low). While our study endorses rACC activity as a strong candidate imaging biomarker in depression, work remains before it might be used in clinical care. The accuracy of the classifier is more modest than will be needed if such a biomarker is to be clinically useful. There is still uncertainty about what underlying function is represented by weaker suppression of rACC activity in depression. Clarifying this mechanism would help to develop better task-related imaging probes that more clearly delineate the subtypes of depression that have better or worse prognoses. While the task we have used is relatively simple, and can be easily implemented on hospital-grade MRI scanners, the cost *v.* benefit of performing such imaging routinely would need to be considered. We have focused only on rACC suppression, which has most often been implicated as a prognostic imaging biomarker for depression. The activity of other brain regions in the context of other task conditions might also be informative, and once they have been more clearly delineated might add to imaging biomarker performance.

Ultimately, the clinical usefulness of prognostic biomarkers such as we have outlined will require validation in prospective clinical trials. For example, patients could be randomised to receive either classifier-guided treatment or treatment-as-usual, along similar lines to the PReDicT trial, which is examining the potential clinical utility of non-imaging parameters (Kingslake et al., 2017). While there is a need for further development of rACC suppression as an imaging biomarker, our results, which extend previous work, suggest a pathway for it to become a tool for clinical decision-making.

## Supplementary material

For supplementary material accompanying this paper visit https://doi.org/10.1017/S0033291721004323. click here to view supplementary material
