# Supplementary material for: Suppressed activity of the rostral anterior cingulate cortex as a biomarker for depression remission
Source: Psychol Med. 2021 Dec 9;53(6):2448–55. doi: 10.1017/S0033291721004323 (PMC10123826; doi:10.1017/S0033291721004323)
Supplement: Supplementary file 1 [file S0033291721004323sup001.docx]

# Supplemental Information

Davey CG, et al. Suppressed activity of the rostral anterior cingulate cortex as a biomarker for depression remission.

**Supplementary Methods.** Expanded Methodology.

**Supplementary Figure 1.** The faces > shapes fMRI contrast (group level result).

**Supplementary Figure 2.** Area under the receiver operator characteristic curve (ROC-AUC) for the fMRI classifier.

**Supplementary Figure 3.** Null distribution for the fMRI classifier after permutation testing.

**Supplementary Table 1.** Table of all clinical predictors entered into the exploratory clinical model for consideration by the elastic net.

## Supplementary Table 2: Baseline characteristics of the trial participants included in fMRI analyses compared to trial participants who were not included.

**Supplementary Table 3.** Changes in symptom severity and rates of remission and response for participants in the YoDA-C trial.

**Supplementary Table 4.** Baseline characteristics of the YoDA-A validation sample.

**Supplementary Table 5.** Changes in symptom severity and rates of remission for participants in the YoDA-A trial.

**Supplementary Table 6.** Activations to the shapes > faces contrast.

## Supplementary Methods

### Inclusion and exclusion criteria

Inclusion criteria were: (1) aged 15 to 25 years; (2) diagnosis of MDD based on the Structured Clinical Interview for DSM-IV Axis I Disorders (SCID-IV) (First, Spitzer, Gibbon, & Williams, 1997); (3) score of 20 or higher on the Montgomery-Åsberg Depression Rating Scale (MADRS) (Montgomery & Asberg, 1979) indicating depression of at least moderate severity; and (4) ability to provide written informed consent (including both adequate intellectual capacity and fluency in the English language). Exclusion criteria were: (1) lifetime or current SCID diagnosis of a psychotic disorder; (2) lifetime or current diagnosis of bipolar I or II disorder; (3) acute or unstable medical disorder that would interfere with treatment; (4) current pregnancy; (5) severe disturbance such that the young person would be unable to comply with the requirements of informed consent or with the study protocol; (6) current treatment with an antidepressant medication for at least two weeks; and (7) previous treatment with fluoxetine that was either ineffective or poorly tolerated.

### Emotional face matching task

The emotional face matching task comprised a shape matching condition and two face matching conditions, containing either fearful or sad facial expressions. In the shape matching condition, participants were required to match the orientation of the shape presented in the top half of the screen to one of the two shapes presented on the left and right in the bottom half of the screen. In the face matching conditions, participants were required to match the sex of the face presented in the top half of the screen with the sex of one of the faces presented on the left and the right in the bottom half of the screen. All of the faces presented in each block, from the Radboud Face Database (Langner et al., 2010), conveyed the same facial expressions. Each block consisted of six trials, with each trial having a duration of 4 seconds. The task-run comprised six blocks of each condition interleaved with 10-second rest-fixation blocks, with order of block presentation counterbalanced between participants (**Figure 1**).


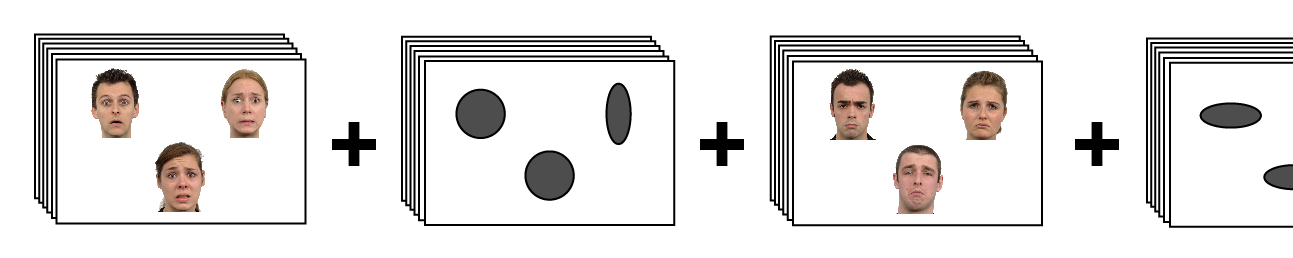


**Figure 1.** The emotional faces task

The task was programmed in Paradigm software and was delivered using MRI-compatible high-resolution goggles (VisuaStim Digital, Resonance Technology Inc.). Participants’ responses were registered with a fORP 4-button response box (Cambridge Research Systems Ltd.), which participants were familiarised with prior to scanning. Participants were required to achieve a minimum accuracy level of 80% across the face matching conditions, indicating that they had correctly understood the instructions and had properly engaged in the task, in order for them to be included in analyses.

### Image acquisition and pre-processing

A 3T General Electric Signa Excite system equipped with an eight-channel phased-array head coil was used in combination with ASSET parallel imaging. The functional sequence consisted of a single shot gradient-recalled echo-planar imaging sequence in the steady state (repetition time, 2 s; echo time, 35 ms; and pulse angle, 90°) in a 23-cm field-of-view, with a 64×64-pixel matrix and a slice thickness of 3.5 mm (no gap). Thirty-six interleaved slices were acquired parallel to the anterior-posterior commissure line with a 20° anterior tilt to better cover ventral prefrontal cortical brain regions. The total sequence time was 10 min 32 s, corresponding to 311 whole brain echo-planar imaging volumes. The first four volumes from each run were automatically discarded to allow for signal equilibration. A T1-weighted high-resolution anatomical image was acquired for each participant to assist with functional time-series co-registration (140 contiguous slices; repetition time, 7.9 s; echo time, 3 s; flip angle, 13°; in a 25.6 cm field-of- view, with a 256 × 256 pixel matrix and a slice thickness of 1 mm).

Imaging data were processed with Statistical Parametric Mapping software (SPM12; Wellcome Trust Centre for Neuroimaging, London, UK) using MATLAB version 9.6 (The MathWorks Inc, Natick, USA). Motion correction was performed by aligning each participant’s time series to the first image using least-squares minimization and a six-parameter rigid-body spatial transformation. The SPM motion fingerprint toolbox (Wilke, 2012) was used to quantify scan-to-scan head motion on the basis of the SPM motion parameters. Participants were excluded if movement exceeded 3 mm (~1 native voxel) mean total scan-to-scan displacement. Subsequently, these realigned functional images were then co-registered to each participant’s respective T1 anatomical scans, which were segmented and spatially normalised to the International Consortium for Brain Mapping template using the unified segmentation approach. The functional images were interpolated to 2 mm isotropic resolution and smoothed with a 5-mm full-width-at-half-maximum (FWHM) gaussian filter.

### References

First, M. B., Spitzer, R. L., Gibbon, M., & Williams, J. B. W. (1997). *Structured Clinical Interview for DSM-IV Axis I Disorders (SCID-I)*. Washington: American Psychiatric Publishing.

Langner, O., Dotsch, R., Bijlstra, G., Wigboldus, D. H. J., Hawk, S. T., & van Knippenberg, A. (2010). Presentation and validation of the Radboud Faces Database. *Cognition & Emotion*, *24*(8), 1377-1388.

Montgomery, S. A., & Asberg, M. (1979). A new depression scale designed to be sensitive to change. *British Journal of Psychiatry*, *134*, 382-389.

Wilke, M. (2012). An alternative approach towards assessing and accounting for individual motion in fMRI timeseries. *Neuroimage*, *59*(3), 2062-2072.


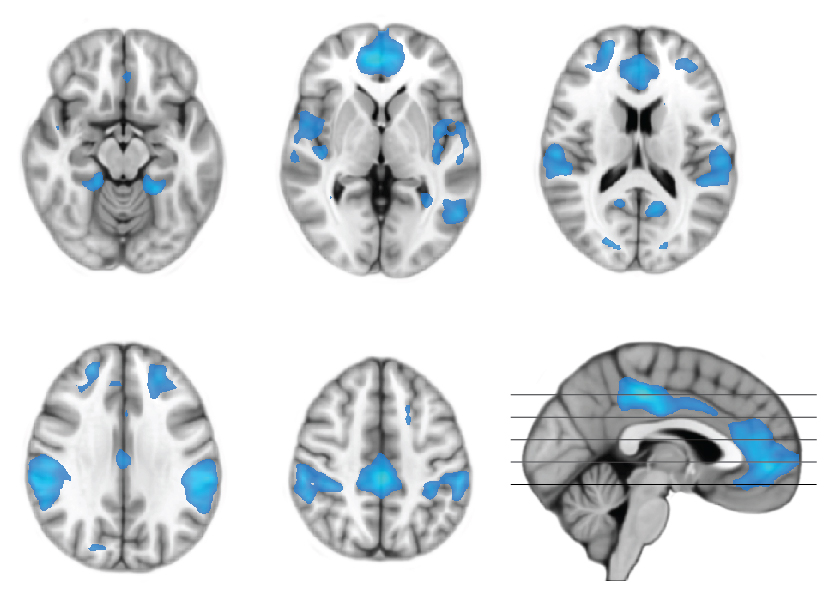


## Supplementary Figure 1. The shapes > faces contrast, showing suppression of activity in the anterior and posterior midline cortices, and other regions, during performance of the face-matching task. The maps are thresholded at whole-brain *P_FDR_* < 0.01.


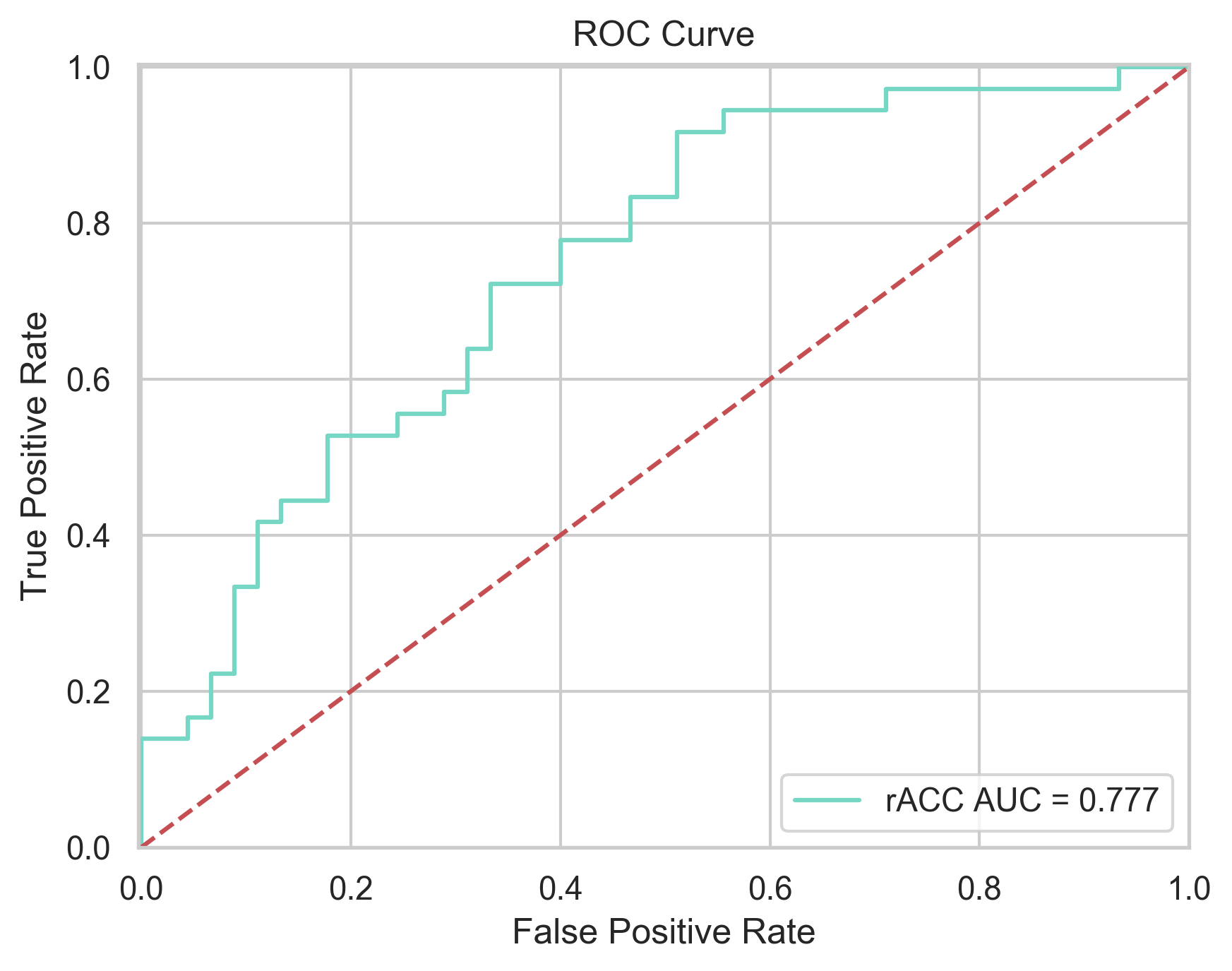


## Supplementary Figure 2. Area under the receiver operator characteristic curve (ROC-AUC) for the fMRI classifier. Trades-offs between true and false positives are observed as the decision threshold for the models underlying probabilities is changed.


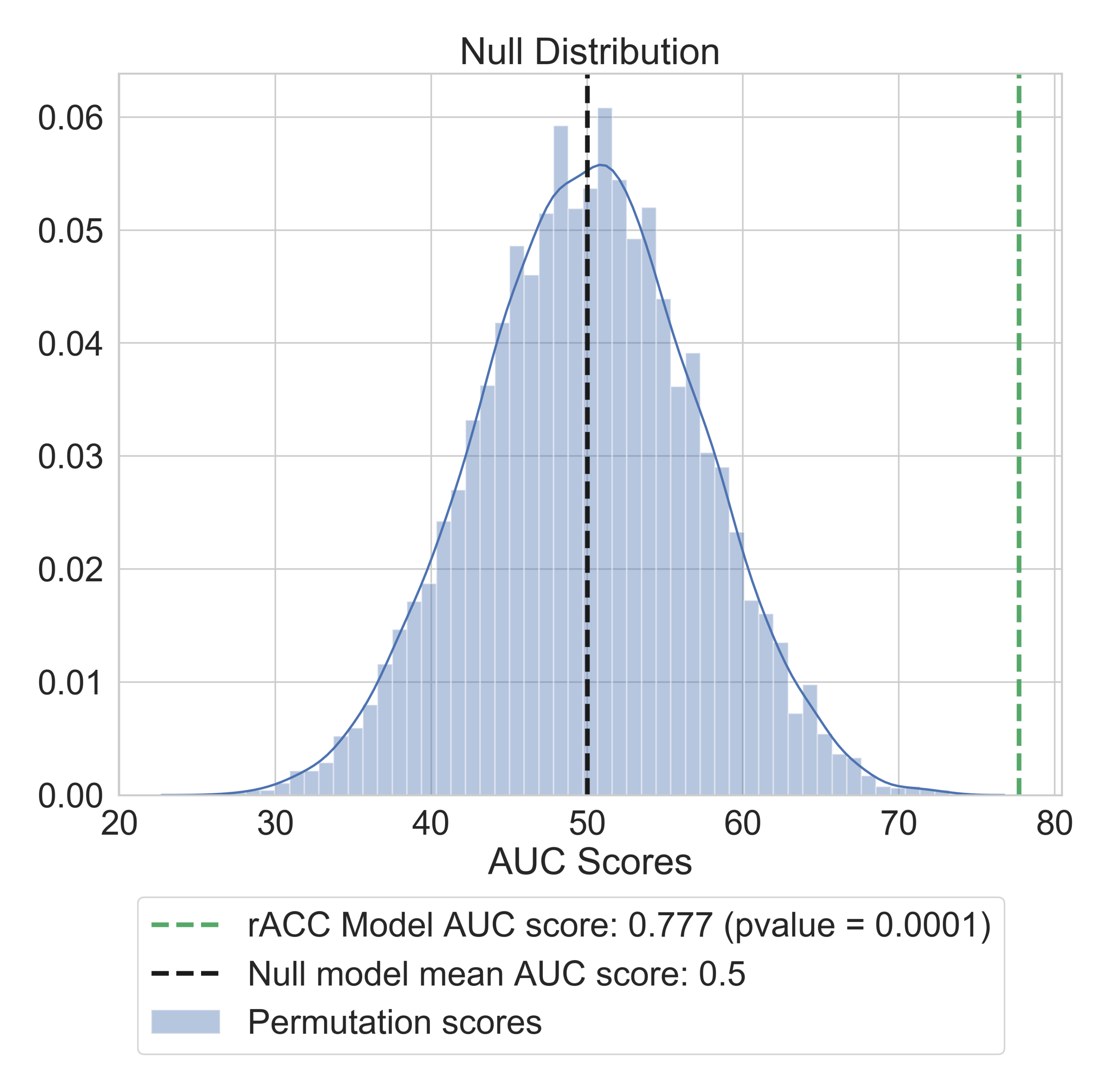


## Supplementary Figure 3. Null distribution for the fMRI classifier after permutation testing (*m* = 10,000). In this procedure, we randomly shuffled class labels (response/non-response) and made predictions with our trained classifier 10,000 times, deriving a null distribution of chance scores for our fMRI model.

## Supplementary Table 1. Table of all clinical predictors entered into the exploratory clinical model for consideration by the elastic net.

| Variable | Description |
| --- | --- |
| *Demographic and clinical variables* | |
| Sex | Birth sex |
| Age_calc | Age in years |
| Site | Recruitment site |
| Ethnicity | Identified ethnicity |
| Occupation | Current occupation |
| Education_level | Maximum achieved level of education |
| Education_years | Number of years of education |
| Primary_language | Language spoken at home |
| AO | Age of MDD onset in years |
| Epi_total | Total number of MDD episodes |
| Duration | Duration of current episode in weeks |
| RxGroup | Treatment allocation: CBT and fluoxetine or CBT and placebo |
| CGI_S | Clinical Global Impression – Severity score |
| Prev_meds | Previous treatment with antidepressant medications |
| *Montgomery-Asberg Depression Scale (MADRS)* | |
| MADRS_total_score | MADRS total score |
| MADRS_Q1 | MADRS Q1: Reports of depressed mood |
| MADRS_Q2 | MADRS Q2: Representing despondency, gloom and despair |
| MADRS_Q3 | MADRS Q3: Feelings of ill-defined discomfort, edginess, inner turmoil |
| MADRS_Q4 | MADRS Q4: Reduced duration or depth of sleep |
| MADRS_Q5 | MADRS Q5: Loss of appetite compared with when-well |
| MADRS_Q6 | MADRS Q6: Difficulties in collecting one's thoughts mounting |
| MADRS_Q7 | MADRS Q7: Difficulty in getting started |
| MADRS_Q8 | MADRS Q8: Reduced interest in the surroundings or activities |
| MADRS_Q9 | MADRS Q9: Thoughts of guilt, inferiority, self-reproach, sinfulness, remorse and ruin |
| MADRS_Q10 | MADRS Q10: The feeling that life is not worth living |
| *Quick Inventory of Depressive Symptomatology (QIDS)* | |
| QIDS_total_baseline | QIDS total score |
| QIDS_Q1 | QIDS Q1: Falling asleep |
| QIDS_Q2 | QIDS Q2: Sleep at night |
| QIDS_Q3 | QIDS Q3: Waking early |
| QIDS_Q4 | QIDS Q4: Sleeping too much |
| QIDS_Q5 | QIDS Q5: Feeling sad |
| QIDS_Q6 | QIDS Q6: Decreased appetite |
| QIDS_Q7 | QIDS Q7: Increased appetite |
| QIDS_Q8 | QIDS Q8: Decreased weight |
| QIDS_Q9 | QIDS Q9: Increased weight |
| QIDS_Q10 | QIDS Q10: Concentration |
| QIDS_Q11 | QIDS Q11: View of myself |
| QIDS_Q12 | QIDS Q12: Thoughts of death or suicide |
| QIDS_Q13 | QIDS Q13: General interest |
| QIDS_Q14 | QIDS Q14: Energy level |
| QIDS_Q15 | QIDS Q15: Feeling slow |
| QIDS_Q16 | QIDS Q16: Feeling restless |
| *Generalized Anxiety Disorder 7-item scale* |  |
| GAD7_total | GAD7 total |
| GAD7_Q1 | GAD7 Q1: Feeling nervous, anxious, or on edge |
| GAD7_Q2 | GAD7 Q2: Not being able to stop or control worrying |
| GAD7_Q3 | GAD7 Q3: Worrying too much about different things |
| GAD7_Q4 | GAD7 Q4: Trouble relaxing |
| GAD7_Q5 | GAD7 Q5: Being so restless that it's hard to sit still |
| GAD7_Q6 | GAD7 Q6: Becoming easily annoyed or irritable |
| GAD7_Q7 | GAD7 Q7: Feeling afraid as if something awful might happen |
| GAD7_difficulty_total | GAD7: How difficult have these made it for you to do your work, take care of things at home, or get along with other people? |
| *Suicidal Ideation Questionnaire (SIQ)* | |
| SIQ_total | SIQ total score |
| SIQ_critical | SIQ critical items subscale |
| *Multidimensional Scale of Perceived Social Support (MSPSS)* | |
| MSPSS_total | MSPSS total score |
| MSPSS_sigother | MSPSS support from significant other subscale |
| MSPSS_family | MSPSS support from family subscale |
| MSPSS_friend | MSPSS: support from friends subscale |
| MSPSS_total | MSPSS total score |
| *Rumination response scale (RRS)* | |
| RRS_total | RRS total score |
| RRS_reflection | RRS reflection subscale |
| RRS_brooding | RRS brooding subscale |
| RRS_deprelated | RRS depression related subscale |
| *Emotional Regulation Questionnaire (ERQ)* | |
| ERQ_reappraisal | ERQ cognitive reappraisal subscale |
| ERQ_suppression | ERQ expressive suppression subscale |
| *Cognitive Emotion Regulation Questionnaire (CERQ)* | |
| CERQ total | CERQ total score |
| CERQ_selfblame | CERQ self-blame subscale |
| CERQ_accept | CERQ acceptance subscale |
| CERQ_ruminate | CERQ rumination subscale |
| CERQ_posrefocus | CERQ positive refocusing subscale |
| CERQ_plan | CERQ refocus on planning subscale |
| CERQ_posreapp | CERQ positive reappraisal subscale |
| CERQ_perspec | CERQ putting into perspective subscale |
| CERQ_catastroph | CERQ catastrophizing subscale |
| CERQ_blameothers | CERQ blaming others subscale |
| *Cognitive Behavior Therapy Skills Questionnaire (CBTSQ)* | |
| CBTSQ_total | CBTSQ total score |
| CBTSQ_behavioural_activation | CBTSQ behavioural activation subscale |
| CBTSQ_cognitive_restructuring | CBTSQ cognitive restructuring subscale |
| *Other measures* | |
| YMRS_total | Young Mania Rating Scale total score |
| BSDS_total | Bipolar Disorder Spectrum Diagnostic Scale total score |
| AUDIT_total | Alcohol Use Disorders Identification Test total score |
| SOFAS_total | Social and Occupational Functioning Assessment Scale total score |
| QLES_total | Quality of Life Enjoyment and Satisfaction total score |
| SASSR_total | Social Adjustment Scale-Self-Report total score |
| WTAR_total | Weschler Test of Adult Reading total score |
| NPOQ_total | Negative Problem Orientation Questionnaire total score |

## Supplementary Table 2: Baseline characteristics of the trial participants included in fMRI analyses compared to trial participants who were not included.

| **Baseline characteristic** | **Descriptive statistic** | **Included** | **Not included** | **Included vs non-included** |
| --- | --- | --- | --- | --- |
|  |  | N = 81 | N = 72 | p-value* |
| Age | mean (SD) | 19.7 (2.7) | 19.4 (2.8) | 0.41 |
| Sex, female | N (%) | 51 (63%) | 39 (54%) | 0.32 |
| MADRS score | mean (SD) | 32.6 (5.5) | 33.2 (5.3) | 0.50 |
| QIDS score | mean (SD) | 17.0 (3.1) | 17.3 (3.4) | 0.55 |
| GAD7 score | mean (SD) | 13.2 (5.3) | 13.6 (5.8) | 0.69 |
| Number of major depressive episodes | median (IQR) | 3 (1–5+) | 2 (1–4) | 0.08 |
| Duration, weeks | median (IQR) | 20 (11–48) | 23 (12–60) | 0.53 |
| Treatment allocation,  CBT and fluoxetine | N (%) | 44 (54%) | 32 (44%) | 0.26 |
| Remitted (MADRS ≤ 12) | N (%) | 36 (44%) | 19/60 (32%) | 0.16 |

*calculated using t-tests for normally distributed variables, Wilcoxon tests for skewed variables, and Fisher’s exact test for categorical variables.

## Supplementary Table 3. Changes in symptom severity and rates of remission and response for participants in the YoDA-C trial.

|  | **CBT+PBO** | | **CBT+FLX** | |  |  |
| --- | --- | --- | --- | --- | --- | --- |
|  | N = 37 | | N = 44 | |  |  |
| **Symptom measures** | change from baseline | 95% CI | change from baseline | 95% CI | mean difference | 95% CI |
| MADRS | -15.7 | -19.0, -12.4 | -14.5 | -17.7, -11.4 | 1.2 | -3.2, 5.5 |
| QIDS | -7.0 | -8.6, -5.4 | -6.9 | -8.4, -5.3 | 0.1 | -2.0, 2.3 |
| GAD7 | -4.0 | -5.6, -2.4 | -5.2 | -6.7, -3.6 | -1.1 | -3.4, 1.1 |
| **Remission / response** | N (%) | | N (%) | | odds ratio | 95% CI |
| MADRS ≤ 12 | 15 (41%) | | 21 (48%) | | 1.3 | 0.5, 3.6 |
| MADRS ≤10 | 12 (32%) | | 18 (41%) | | 1.4 | 0.5, 4.0 |
| MADRS ≤ 7 | 8 (22%) | | 12 (27%) | | 1.4 | 0.4, 4.4 |
| MADRS ≤ 50% | 18 (49%) | | 21 (48%) | | 1.0 | 0.4, 2.5 |

## Supplementary Table 4. Baseline characteristics of the YoDA-A validation sample

| **Baseline characteristic** | **Descriptive statistic** | **Remission** | **Non-remission** | **Remission vs non-remission** |
| --- | --- | --- | --- | --- |
|  |  | N = 13 | N = 12 | p-value* |
| Age | mean (SD) | 19.7 (2.7) | 20.8 (2.6) | 0.30 |
| Sex, female | N (%) | 3 (23%) | 6 (50%) | 0.22 |
| MADRS score | mean (SD) | 30.4 (3.9) | 35.0 (6.2) | 0.04 |
| QIDS score | mean (SD) | 15.6 (5.5) | 20.0 (3.5) | 0.03 |
| GAD7 score | mean (SD) | 11.7 (6.5) | 14.7 (4.6) | 0.20 |
| Number of major depressive episodes | median (IQR) | 3 (1–5) | 2 (1–4) | 0.59 |
| Duration, weeks | median (IQR) | 23 (5–38) | 23 (6–70) | 0.66 |
| Treatment allocation |  |  |  |  |
| placebo | N (%) | 2 (15%) | 3 (25%) |  |
| rosuvastatin | N (%) | 8 (62%) | 5 (42%) |  |
| aspirin | N (%) | 3 (23%) | 4 (33%) | 0.65 |

## Supplementary Table 5. Changes in symptom severity and rates of remission for participants in the YoDA-A trial

|  | **Placebo** | | **Rosuvastatin** | | **Aspirin** | |
| --- | --- | --- | --- | --- | --- | --- |
|  | N = 5 | | N = 13 | | N = 7 | |
| **Symptom measures** | change from baseline | 95% CI | change from baseline | 95% CI | change from baseline | 95% CI |
| MADRS | -13.2 | -21.8, 5.3 | -18.8 | -22.1, -13.6 | -14.1 | -30.8, -6.9 |
| QIDS | -4.4 | -10.6, 0.0 | -7.6 | -11.0, -4.3 | -7.7 | -17.4, -3.5 |
| GAD7 | -5.0 | -9.1, -0.9 | -5.3 | -9.3, -2.1 | -6.7 | -12.9, -2.9 |
| **Remission**  N (%) | 2 (40%) | | 8 (62%) | | 3 (43%) | |

## Supplementary Table 6. Activations to the shapes > faces contrast (i.e., areas suppressed during the face-matching task).

| **Region** | **Cluster size** | **Peak voxel coordinate**  **[*x y z*]** | ***z*-value** |
| --- | --- | --- | --- |
| PCC | 1637 | 0 -28 42 | 7.6 |
| Left inferior parietal lobule | 2745 | -58 -32 36 | 7.6 |
| Left temporo-occipital cortex | 412 | -28 -50 -6 | 7.4 |
| Right temporo-occipital cortex | 343 | 26 -48 -8 | 7.3 |
| Rostral ACC | 2380 | 6 48 0 | 7.2 |
| Right lateral occipital cortex | 228 | 22 -82 20 | 6.5 |
| Right inferior parietal lobule | 3713 | 36 -32 40 | 6.4 |
| Left posterior temporal cortex | 273 | -52 -60 -4 | 6.4 |
| Right frontal pole | 350 | 22 48 28 | 5.4 |
| Left posterior insula | 280 | -40 -14 -4 | 5.3 |
| Left precuneous | 237 | -12 -60 18 | 5.3 |
| Left lateral occipital cortex | 130 | -18 -84 18 | 5.1 |
| Left frontal pole | 616 | -24 40 30 | 5.1 |
| Right posterior insula | 153 | -58 -6 4 | 4.3 |

Contrasts were thresholded at whole-brain *P_FDR_* < 0.01, with reporting of clusters > 80 voxels.
